# Different approaches for assessing sperm function

**DOI:** 10.21451/1984-3143-AR2018-122

**Published:** 2020-05-22

**Authors:** Diego Bucci, Marcella Spinaci, Giovanna Galeati, Carlo Tamanini

**Affiliations:** Department of Veterinary Medical Sciences, Via Tolara di Sopra, 50 40064 Ozzano dell’Emilia, Bologna, Italy.

**Keywords:** sperm function, multiparametric approach

## Abstract

Different approaches can be used to assess sperm function in different conditions, i.e. sperm storage, freezing-thawing or activation by induction of capacitation and acrosome reaction.

In this review we will focus on the assays routinely performed in our laboratories, giving a literature support to critically analyse different approaches. In fact, researchers usually tend to look for the “one shot” parameter that could explain itself a specific process; it is our conviction that a multiparametric approach is still more valid, as some changes in sperm function are very complex and could be explained only by operating in different ways.

Sperm motility, the most evident sperm characteristic, should be assessed by computer-aided sperm analysers that permit an objective evaluation of the motility and its kinematic parameters. Commercial and open source instruments are available and could be profitably used together with specific statistical approaches. The use of microscopy, and particularly fluorescent microscopy, could be a very useful tool to assess different parameters in sperm cells both by fluorophores that give indication of a determined function, and by immunolocalization of proteins, that permits the discover of new features or to explain particular sperm functions. The same substrates could be used also in flow cytometry: the difference is that it permits to study wider sperm populations (and their sub-population distribution). Flow cytometry is undergoing a very wide use in spermatology and technical and experimental rigor is needed to obtain reliable results. Metabolic assessment of sperm features, particularly energetic supply, ATP formation and other enzyme activities, could represent a very important challenge to acquire new information and complete/integrate those derived from other techniques. Finally, functional assays such as oocyte binding and *in vitro* fertilization, represent a very strong tool to assess sperm function *in vitro*, as they could evidence the functional intactness of some pathways.

## Introduction

Spermatozoa are cells specifically programmed to deliver male genetic material to the female gamete, thus permitting fertilization and born of a new individual.

To be able to achieve this goal, spermatozoa should undergo numerous functional adaptations from the time they are ejaculated, passing through male genital tract, then female one, to reach the fertilization site (usually the ampulla of the oviduct; Suarez and Pacey, 2006).

*In vitro* assays to assess different sperm functions are, at present, a very important tool to understand and discover the pathways implicated in sperm function and their changes. Different approaches are used nowadays to assess sperm features *in vitro*: sperm motility analysis systems (Amann and Katz, 2004; Hoogewijs *et al*., 2012; Boryshpolets *et al*., 2013; Amann and Waberski, 2014), morphology (Morrell, 2006; Love, 2011), fluorescent microscopy (Mattioli *et al*., 1996; Gravance *et al*., 2000; Flesch *et al*., 2001; Guthrie *et al*., 2008; Ortega Ferrusola *et al*., 2009; Satorre *et al*., 2009; Kim *et al*., 2011), protein analysis (Spinaci *et al*., 2005b; Krisfalusi *et al*., 2006; Gadella, 2008; Flores *et al*., 2011; Kumaresan *et al*., 2011), flow cytometry (Didion *et al*., 2009; Martìnez-Pastor *et al*., 2010; Petrunkina and Harrison, 2010; Hossain *et al*., 2011; Gürler *et al*., 2015; Barrier Battut *et al*., 2016; Battut *et al*., 2017), metabolic assay (Albarracín *et al*., 2004; Storey, 2008; Bucci *et al*., 2011; Gibb and Aitken, 2016; Rodríguez-Gil and Bonet, 2016); sperm oocyte interaction (Funahashi *et al*., 1997; Sinowatz *et al*., 2003; McPartlin *et al*., 2008; Mugnier *et al*., 2009; Balao da Silva *et al*., 2013; Plaza Dávila *et al*., 2015).

The present review was aimed at presenting some of the most important approaches in mammalian sperm function analysis, with particular focus on our laboratory experience and at furnishing a critical view of the different parameters examined.

## Sperm motility assessment

Sperm motility is one of the most evident features of spermatozoa, even if its significance is sometimes misestimated (Suarez and Pacey, 2006): spermatozoa are mainly passively transported in the female genital tract, while their motility is especially required for reaching the egg and during zona pellucida penetration (Schmidt and Kamp, 2004; Suarez, 1996, 2008; Boryshpolets *et al*., 2015).

Subjective motility evaluation is commonly used to assess the quality of liquid stored and cryopreserved semen but sperm motility is at present studied by mean of computer assisted sperm analysers (CASA systems) (Mortimer, 2000; Amann and Waberski, 2014). To date, the most common CASA systems are mid to high-cost systems; this prevented a widespread diffusion of this technology, that is specifically used in research centres, university labs or high-level sperm producing centres.

Motility analysis permits to determine the total motility of a determined sample, the progressive one, and other so-called sperm kinematic parameters (Mortimer, 2000; Schmidt and Kamp, 2004; Amann and Waberski, 2014) such as velocities (curvilinear, VCL; average, VAP; straight-line, VSL) straightness and linearity, amplitude of lateral head displacement and beat cross frequency. The approach in sperm motility study could be different, depending on the output data and the needs of the researcher.

In fact the simplest but profitable approach is to analyse sperm motility and use the average results given from the machine; this is a powerful analysis, as it is usually indicated to analyse at least 1000 sperm cell, thus furnishing a strong representation of the average motility features of a given sample (Mortimer, 2000; Contri *et al*., 2010; Hoogewijs *et al*., 2012; Amann and Waberski, 2014;). This approach could be useful in comparing ejaculates of different males from the same species, or differently treated sperm samples (i.e. freshly ejaculates sperm vs frozen-thawed ones). 

A more complex approach is the multiparametric statistical analysis of CASA output. In this case, single sperm kinematic features are studied to perform a subpopulation study: usually a cluster analysis followed by a multiparametric regression (Quintero-Moreno *et al*., 2004; Schmidt and Kamp, 2004; Martínez-Pastor *et al*., 2011; Bucci *et al*., 2018). This combination of statistical techniques permits to delineate features that could be masked by the average parameters approach: in a determined sperm sample, various cell subpopulations are present and display different features. This is why the multiparametric approach is more powerful and profitable for studying motility patterns, kinematic features and particular differences also among samples.

In the last years, growing interest is dedicated to CASA systems based on open source software (Wilson-Leedy and Ingermann, 2007; Boryshpolets *et al*., 2013; Giaretta *et al*., 2017): these alternatives to commercial systems allow a wider use of the technology and could also give a push to the standardization of good laboratory practices for this kind of analysis.

## Sperm analysis by epifluorescence microscopy

A very precious tool for studying morphological and functional characteristics of sperm cells is, obviously, the microscope. In this review we do not approach standard microscopic evaluation, that represented a golden standard of sperm evaluation in the past (i.e. morphology) (Love, 2011) but we will focus on sperm analysis by fluorescent probes or fluorochrome-conjugated antibodies, that permit an analysis of different sperm conditions and functions by a direct visualization of the single sperm cell. 

Several fluorescent probes are used in spermatology (Silva and Gadella, 2006), most of which regard sperm membrane integrity (SYBR green 14; propidium iodide; sytox green) (Maxwell and Johnson, 1997), acrosomal membrane integrity (FITC or TRITC- conjugated Pisum sativum agglutinin and Peanuts agglutinin) (Mari *et al*., 2010), mitochondrial activity (JC1) (Gravance *et al*., 2000; Giaretta *et al*., 2014) calcium storage sites (chlortetracycline - CTC, fluo3 and fluo 4) (Green and Watson, 2001; Bucci *et al*., 2012); membrane destabilization (Annexin V FITC-conjugated) (Spinaci *et al*., 2005a); caspase activation (VAD-fmk FITC-conjugated) (Vallorani *et al*., 2010); actin cytoskeleton reorganization (phalloidin TRITC-conjugated) (Brener, 2002); DNA fragmentation (Halomax ®) (De Ambrogi *et al*., 2006; Alkmin *et al*., 2013). These are some examples that clearly explain how wide the use of fluorescent microscopy in spermatology could be. The main approach followed by researchers when using fluorescent probes, is to visualize single sperm and understand if the probe stains the target or not. The evaluation is performed on a smeared sample and a proper number of cells (usually at least 200) is examined. In this way, this technique allows to perform both a description study (to delineate which sperm cell compartments are stained by a specific probe) and a sub-population study, as it is possible to distinguish the probe positive subpopulation from the negative one. When using more than one probe mixed together (i.e. JC1+PI+SYBR green 14), the subpopulation analysis is enriched in different classes (i.e. live cells with/without active mitochondria; dead cells with/without active mitochondria)

Furthermore, this kind of approach permits to examine particular features of some organelles and their specific activation patterns (Ramió-Lluch *et al*., 2011b).

The most interesting application of fluorescent microscopy regards immunolocalization studies using fluorophore-conjugated antibodies. Several studies from our and many other laboratories (Maccarrone *et al*., 2005; Spinaci *et al*., 2005b; Jones *et al*., 2008; Bucci *et al*., 2010b, 2010a; Flores *et al*., 2010; Bucci *et al*., 2011, 2012; Ramió-Lluch *et al*., 2012; Spinaci *et al*., 2013, 2014) used antibodies against a specific protein or protein residue to detect the presence of the specific proteins in sperm cells as well as to describe their localization and eventual relocalization at different functional moments such as capacitation.

Moreover, this kind of approach is fundamental in discovering new features of the sperm cell and eventually new proteins (or at least proteins that are not known to be expressed in the spermatozoon) that may play interesting and surprising roles (Flores *et al*., 2010; Ramió-Lluch *et al*., 2012; Spinaci *et al*., 2014).

## Protein analysis

Analysis of protein expression is of fundamental importance in trying to investigate sperm function, as new proteins could be discovered in the mature sperm cell and their function could be elucidated. Several studies demonstrated the presence of proteins by both visual inspecting technique (microscopy) and protein analysis such as western blotting (Flores *et al*., 2008; Bucci *et al*., 2010b, 2010a; Flores *et al*., 2011; Spinaci *et al*., 2014). The latter is usually considered the most specific technique to effectively recognize a determined protein, as the possibility to get false positive results is significantly lower if compared with immunofluorescence technique.

Western blotting analysis also permits to quantify the amount of a determined protein; this approach could be of fundamental importance in understanding some physiological processes or changes induced by sperm biotechnical procedures(Spinaci *et al*., 2005b; 2006). Again, this technique, coupled with immuolocalization of the protein of interest, could give interesting and fundamental information regarding the amount as well as possible changes in the site of expression of a given protein. 

In addition, the use of specific antibodies could be very useful in detecting changes in the quantity of activated proteins in a determined functional state (i.e. capacitation or acrosome reaction or post-thawing). This is the case of studies regarding regulatory proteins of determined intracellular pathways (Harayama *et al*., 2004; Maccarone *et al*., 2005; Grasa *et al*., 2009; Ramió-Lluch *et al*., 2011a,b; Bucci *et al*., 2012; Gonzalez-Fernandez *et al*., 2012; Hurtado De Llera *et al*., 2013; Gonzalez- Fernandez *et al*., 2013; Yeste *et al*., 2014); in the reported examples specific antibodies against proteins/activated proteins were used to determine the status of activation of specific pathways and/or of the whole sperm cell.

The central role of proteins in mammalian cell processes should be taken into account also in spermatology; in this way, several proteins have been suggested as specific indicators of determined functions (Spinaci *et al*., 2005b; Pinart *et al*., 2015), even if caution should be given to this kind of approach. It is a common will of researchers to find the “one-man band” parameter, able to describe, alone, an entire process. Anyway, it is not so easy to detect such a parameter nor it is always explicative of the entire process.

## Flow cytometry

In the last two or three decades, flow cytometry gained a very high importance in spermatology: the diffusion of numerous cytometers as well as their high performance in analysing cells has attracted the attention of many researchers.

Flow cytometry mainly bases on the possibility to analyse single cells passing through a liquid flow by exciting them with specific lasers and reading the response of the cell. Fluorochromes play an important role in this system as, choosing the right ones, permit to study different functions/features of the cell (Hossain *et al*., 2011).

Usually, flow cytometry (similarly to what seen in epifluorescence microscopy) permits to determine different populations of sperm cells, on the basis of their positivity to a determined stain (Hossain *et al*., 2011), also combining two or more dyes (Martìnez-Pastor *et al*., 2010; Robles and Martínez-Pastor, 2013).

In spermatology numerous assays can be performed by flow cytometry (Guthrie and Welch, 2007; Martìnez-Pastor *et al*., 2010; Hossain *et al*., 2011; Robles and Martínez-Pastor, 2013) thus allowing a very wide range of parameters to be assessed by this technique. 

Before giving some examples, some important points need to be highlighted. After some years of experience in working on flow cytometry, we have noticed that sometimes literature lacks some important information regarding the technique: the experimental design as well as the technical characteristics of the flow cytometer and the setting used in the analysis are completely missing. It should be recommendable to have a rigorous approach in describing materials and methods, as reported in (Lee *et al*., 2009).

Another important technical remark regards the possibility to overestimate some sperm subpopulations, namely those that are negative for determined staining; in this case, in fact, the negative subpopulation could contain some non-cellular particles (cell acrosomes, tails; non-sperm particles) that could be detected by instrument. To overcome this technical gap, (Petrunkina *et al*., (2010) and Petrunkina and Harrison (2010) have developed a specific technique as well as a mathematic formula to avoid big mistakes in the subpopulation estimation.

Flow cytometry has been used in different species (Maxwell and Johnson, 1997; Rijsselaere *et al*., 2005; Pinart *et al*., 2015; Battut *et al*., 2017) to determine both sperm quality and function.

The technique permits to estimate membrane integrity (Pinart *et al*., 2015; Bucci *et al*., 2018), mitochondrial function and ROS production (Gravance *et al*., 2000; Koppers *et al*., 2008; Gibb *et al*., 2015; Gürler *et al*., 2015; Bucci *et al*., 2018), lipid peroxidation (Aitken *et al*., 2007; Ortega Ferrusola *et al*., 2009), sperm capacitation (Rathi *et al*., 2001; Piehler *et al*., 2006; Martìnez-Pastor *et al*., 2010; Hossain *et al*., 2011); oxidative status (Gibb *et al*., 2014; Giaretta *et al*., 2015).

The use of fluorochrome-bound antibodies against specific proteins or activated proteins (i.e.phosphorilated ones) is of actual importance (Piehler *et al*., 2006); it should be noted that, in the case of flow cytometry, the instrument output when using antibodies could be related to the presence/absence of the determined protein and, partially, on the intensity of the signal (that could be related to the amount of the protein). This technique does not give any information on the localization of the protein within the cell, even if some new cytometers permit also the visual inspecting of the cells passing through the flow.

At present, multiparametric analysis are possible, and the development of newest cytometers with more lasers and optical channels permits the contemporary assessment of different parameters thus giving a multiparametric output that is extremely precious for determining specific cell functional statuses (i.e. capacitation).

## Sperm metabolism

A consistent part of sperm function we can analyse by the techniques above described represents the final evidence of numerous metabolic processes that characterize sperm cell. Thinking of capacitation, for example, different approaches to determine the effective status of the cell are possible (calcium relocation; tyrosine phosphorylation; motility changes; membrane scrambling and so on, see above) but other functions of the cell are beneath these effects.

Sperm cells metabolism has been studied in different species and under different aspects: the first, and probably the most important one is energy supply and production. For energy substrate supply, different researchers analysed the presence and function of sugar transporters (Burant, 1992; Angulo *et al*., 1998; Sancho *et al*., 2007; Bucci *et al*., 2010a, 2010b, 2011). These studies, were coupled with others determining the internal energetic pathways of different sugars in different species (Ballester *et al*., 2000; Rigau *et al*., 2001, 2002; Marin *et al*., 2003; Albarracín *et al*., 2004; Fernández-Novell *et al*., 2004; Mukai and Okuno, 2004; Medrano *et al*., 2005; Urner and Sakkas, 2005; Ford, 2006; Medrano *et al*., 2006; Rodriguez-Gil, 2006; Terrell *et al*., 2011; Rodríguez-Gil and Bonet, 2016). All together these studies delineated the metabolic strategies in energy obtainment in boar, dog, felids and horse spermatozoa, defining different metabolic strategies of sperm cells on the basis of their ability to better use hexoses.

Recently more knowledge was acquired on horse sperm metabolism, in particular regarding sperm function and capacity to overcome specific metabolic situations induced by sperm preservation *in vitro* (Gibb *et al*., 2015; Varner *et al*., 2015; Gibb and Aitken, 2016).

Mitochondrial function has also been focused and better delineated in recent years (Davila *et al*., 2015; Peña *et al*., 2015) by the use of specific inhibitors of different mitochondria compartments (complex I or III). At present, we are performing studies ( unpublished data) regarding the mitochondrial function in boar sperm cells. Using different specific inhibitors for complex I, II, II, V, uncoupling agents and glucose agonists, we are trying to define the metabolic role of mitochondria by ATP production, oxygen consumption analysis as well as relating to sperm function as motility and membrane stability, deepening some previous studies by others (Ramió-Lluch *et al*., 2014).

The metabolic activity of sperm cells could also be evaluated by measuring enzymatic activity of different enzymes (Glogowski *et al*., 2002; Turner and McDonnell, 2003; Pesch *et al*., 2006; Cocchia *et al*., 2011; Kareskoski *et al*., 2011; Pinart *et al*., 2015; Bucci *et al*., 2014, 2017, 2016,) that could play focal roles in different sperm functions.

## Sperm-oocyte interaction assays

Sperm-oocyte interaction is a naturally occurring event when spermatozoa have almost finished their travel along the female genital tract (Suarez and Pacey, 2006). Usually, mammalian spermatozoa encounter the oocyte after having undergone some profound membrane, biochemical and functional modifications known as “capacitation” (Gadella, 2008; Tsai *et al*., 2010; Leahy and Gadella, 2011).

After penetration through cumulus ooforus, the first interaction between spermatozoon and oocyte is sperm binding to zona pellucida; thereafter spermatozoa undergo acrosome reaction, penetrate the zona and the plasma membrane and finally the sperm nucleus decondenses and forms the male pronucleus. This events’ succession is reproducible also *in vitro*, via binding assays as well as through *in vitro* fertilization (IVF) assays. 

Oocyte binding is a very useful tool to evaluate sperm function: spermatozoa are submitted to different stimuli (i.e. capacitation, freezing; use of specific substances) and then their capacity to bind to the zona pellucida is assayed (Sinowatz *et al*., 2003; Bucci *et al*., 2016, 2017; Spinaci *et al*., 2017). The binding assay could be very important to assess sperm function in those species in which IVF is not reliably successful, such as horse (Balao da Silva *et al*., 2013; Plaza Dávila *et al*., 2015; Bucci *et al*., 2017).

In some cases it is possible to use the homologous oocyte binding assay (Sinowatz *et al*., 2003; Bucci *et al*., 2017; Spinaci *et al*., 2017), that permits a more stable binding between sperm and oocyte. In our studies we performed homologous oocyte binding in pig (Spinaci *et al*., 2017), horse (Bucci *et al*., 2017) and dog (unpublished data). When the amount of oocytes needed is difficult to obtain (usually in horse as well as in dog), the heterologous oocyte binding could be profitably used to assess sperm function (Balao da Silva *et al*., 2013; Plaza Dávila *et al*., 2015; Bucci *et al*., 2016).

IVF trials to assess sperm function are focused on the ability of a (capacitated) spermatozoon to fertilize the egg and to actively penetrate the oocyte. In these assays oocytes are merely a kind of experimental substrate used to determine sperm function. Many examples could be furnished (Bucci *et al*., 2014; Gadani *et al*., 2017; Bucci *et al*., 2018; Spinaci *et al*., 2018), in which usually three parameters are considered: penetration rate (number of oocytes penetrated by spermatozoa %), monospermy rate (number of oocytes penetrated by only one spermatozoon %) and efficiency (number of monospermic oocytes /number of penetrated oocytes %).

This kind of data permits a wider understanding of the effect of a treatment on sperm capacitation, or to delineate whether a specific treatment (i.e. freezing, supplementation to IVF medium) could either positively or negatively affect sperm functionality.

## Concluding remarks

Different approaches have been developed to carry out a wide and exhaustive analysis of sperm quality parameters. The information gained from the different approaches is of utmost importance for studying sperm physiology, function and intrinsic features. Different approaches using various techniques to assess specific functional moments of the sperm cell (i.e. capacitation, acrosome reaction) permit to elucidate various pathways underlying a specific function and to have a wider view on a specific event. Researchers usually look for the “on-off” parameters for specific events, but studies on spermatology during the last decades demonstrated that a multiparametric approach is preferable as some events of sperm life cannot be explicated by only one parameter.

Finally, an important remark to be kept in mind: at present multiparametric analysis is not effective to predict *in vivo* male fertility in a reliable manner. Various parameters have been demonstrated to be more linked to sperm fertility (i.e. DNA integrity; motility features), but no definitive parameters have been determined and fixed as a golden standard to assess male fertility *in vivo* This could be one of the challenges for future studies in spermatology, to improve male reproductive performances in animals and also to permit an early diagnosis of infertility, also in man, thus giving the possibility to intervene precociously in treating such problems. 
